# Quality of life among Sudanese patients with multiple sclerosis in Khartoum state using (MSQoL-54) questionnaire

**DOI:** 10.1186/s13104-019-4565-9

**Published:** 2019-08-22

**Authors:** Etedal Ahmed Abu Elbasher Ibrahim, Alsadig Gassoum, Israa El Imam IshagAgib

**Affiliations:** 1 Alneelain University, Khartoum, Sudan; 2 The National Centre for Neurological Science, Khartoum, Sudan; 3 Almadain College for Medical Sciences and Technology (ACMST), Khartoum, Sudan

**Keywords:** Quality of life, Multiple Sclerosis, MSQOL-54, Sudan

## Abstract

**Objective:**

To assess the quality of life, physical and mental factors, determine association between age, education status, disease duration and MSQOL-54 physical and Mental Health Composite scores among Sudanese patients with multiple sclerosis. This cross-section study was conducted among 32 MS patients at the National center for Neurological Science, Khartoum from January 2017 to June 2018. Using Multiple sclerosis Quality of life (MSQoL-54) questionnaire.

**Results:**

Thirty two MS patients were participated in this study, 30 (94%) were females and 2 (6%) were males, 25 (78.1%) were found in age group < 40 years overall score of MSQoL-54 was 66.4 ± 21.9, Physical Health Composite (PHC) was 71.6 ± 21.3 and Mental Health Composite (MHC) was 61.2 ± 22.4. This study indicated that, non-graduated, advanced age and longer disease duration was significantly associated with poor Physical Health Composite and Mental Health Composite.

**Electronic supplementary material:**

The online version of this article (10.1186/s13104-019-4565-9) contains supplementary material, which is available to authorized users.

## Introduction

Multiple sclerosis is a demyelinating disease and the most common immune-mediated disorder affecting the central nervous system [1]. The disease is common among young usually female of child bearing age in white population, temperate climate region and high socio-economic community and thought to be rare in North African countries like Sudan. The disease is a common cause of disability among our population, so measurement of Quality of life, both health and mental composite are vital for the medical care, rehabilitation and nursing. Furthermore this is the first study to determine the QoL in MS Sudanese patients. Several studies have identically shown, that patients with multiple sclerosis experience lower Quality of life compared to healthy control group [2]. Fatigue is the most common symptom [2]. In addition to the chronic nature of the disease, lack of prognosis and definitive therapy, cause several psychological symptoms among which depression, anxiety and stress are the most common [3]. Specific symptoms include double vision, blindness in one eye, muscle weakness, trouble with sensation, or trouble with coordination. MS takes several forms, with new symptoms either occurring in isolated attacks (relapsing forms) or building up over time (progressive forms). Between attacks, symptoms may disappear completely; however, permanent neurological problems often remain, especially as the disease progress [4].

## Main text

### Methods

This descriptive cross-section study was conducted from January 2017 to June 2018. Thirty two (32) patients aged 18+ years were diagnosed with multiple sclerosis clinically and radiologically according to modified Macdonald criteria were recruited and investigated by using Multiple sclerosis Quality of life MSQoL-54 questionnaire. The sub scales are: physical function, role limitations-physical, role limitations-emotional, pain, emotional well-being, energy, health perceptions, social function, cognitive function, health distress, overall quality of life, and sexual function. Other neurological diseases, serious cardiovascular, orthopedic or other disability were excluded from the study. Data were analyzed by the Statistical Package for Social Science (SPSS), version 21. Chi square test. P value of < 0.05 was considered to be statistically significant. Descriptive statistics was used to calculate the mean and the standard deviation of the total scores and the sub-scales of the MSQoL-54 tool. Psychometric properties the MSQoL-54 12 subscales show good internal consistency with Cronbach Alpha ranging from 0.75 to 0.96. Test–retest reliability is good with interclass correlation co-efficiency ranging from 0.66 to 0.96. There is evidence for the validity of the questionnaire. The questionnaire in this study showed associations with MS symptom severity, level of ambulation, employment limitations due to health problems and hospital admission (Additional file 1) [5].

Ethical approval was obtained from National Center for Neurological Science, Khartoum, Sudan.

### Results

A total of 32 MS patients, 30 (94%) were females and 2 (6%) were males most of them 25 (78.1%) were found in age group less than 40 years. Concerning to the marital status, one-half 16 (50%) were married, 12 (37.5%) were singles, 3 (9.4%) were divorced and one patient (3.1%) was widower.

Regarding to the origin, 17 (53.1%) of the patients were from north, 10 (31.3%) from east, 4 (12.5%) from west and one patients from south. Most of the patients 27 (84.4%) were university educated, 3 (9.4%) primary educated and 2 (6.3%) were secondary educated. The majority of the patients 17 (53.1%) had disease duration from 5 to 10 years, 11 (34.4%) less than 5 years and 4 (12.5%) of them had MS duration for more than 10 years. The overall score of MSQoL-54 among patients was 66.4 ± 21.9, Physical Health Composite (PHC) was 71.6 ± 21.3 and Mental Health Composite (MHC) was 61.2 ± 22.4. In comparison between the mean score of MSQoL-54 subsets (PHC and MHC) in MS patients, Descriptive statistics was used to calculate the mean and the standard deviation of the total scores and the sub-scales of the MSQoL-54 tool. Physical Health Composite (PHC) and Mental Health Composite (MHC) was significantly associated with the ages of the patients as; the younger patients (< 40 years) were significantly had highest mean score in both PHC (78.5 ± 11.5; P = 0.000) and MHC (69.6 ± 13.3; P = 0.001) than other groups (Tables 1, 2). Moreover, Physical Health Composite (PHC) and Mental Health Composite (MHC) was significantly associated with educational levels of the patients as; the university educated patients (< 40 years) were significantly had highest mean score in both PHC (82.3 ± 17.7; P = 0.000) and MHC (75.6 ± 17.7; P = 0.000) than other groups (Table 3). Similarly, Physical Health Composite (PHC) and Mental Health Composite (MHC) was significantly associated with the MS duration of the patients as; patients with duration < 5 years were significantly had highest mean score in both PHC (70.2 ± 14.1; P = 0.000) and MHC (75.3 ± 13.6; P = 0.000) than other groups (Additional file 2: Table S1).

**Table 1:** The mean and standard deviation of MSQoL-54 among Sudanese MS patients in Khartoum state, 2018 (N = 32)

	Mean	SD
Physical Health Composite (PHC)	71.6	21.3
Mental Health Composite (MHC)	61.2	22.4
Overall score	66.4	21.9

**Table 2:** The comparison between MSQoL-54 subsets (PHC and MHC) in MS patients in Khartoum state regarding to the age (N = 32)

Age (years)	Physical Health Composite (PHC)	Mental Health Composite (MHC)
	Mean (SD)	Mean (SD)
< 40	78.5 (11.5)	69.6 (13.3)
40–60	62.6 (17.1)	57.7 (18.0)
> 60	56.1 (15.7)	50.1 (17.7)
P value	0.000*	0.001*

ANOVA test was used

* P value is significant (< 0.05)

**Table 3:** The comparison between MSQoL-54 subsets (PHC and MHC) in MS patients in Khartoum state regarding to the educational levels (N = 32)

Educational levels	Physical Health Composite (PHC)	Mental Health Composite (MHC)
	Mean (SD)	Mean (SD)
Primary	66.9 (10.3)	56.4 (15.0)
Secondary	71.3 (16.1)	63.9 (12.0)
University	82.3 (17.7)	75.6 (17.7)
P value	0.000*	0.000*

ANOVA test was used

* P value is significant (< 0.05)

### Discussion

Our study is the first comprehensive attempt to provide an estimate of quality of life in multiple sclerosis patients. In this study, most of the patients were young less than 40 years (76.7%). And this is similar to other studies done by Berer K et al. and Lozano et al. which stated that, the age of multiple sclerosis was found to be between the ages of 15 to 45 years [1, 6]. Our study showed that, the Quality of life overall mean score was 66.4 ± 21.9, Physical Health Composite was 71.6 ± 21.3 and Mental Health Composite was 61.2 ± 22.4. This was similar to the findings reported by Algahtani et al. [7] who found that, quality of life of MS patients measured 73.87 ± 23.41 associated with significant effects on the patient’s life. On the other hand, Sara et al. [8] noticed that, males had significantly higher scores compared to females in both physical and mental domains of MSQOL-54 (P < 0.001). Furthermore, in this study, secondary graduates with age less than 40 years, with longer disease duration was significantly associated with poor Physical Health Composite and Mental Health Composite in MSQOL-54 tool subsets (P < 0.005). In addition to that, younger primary school and illiterates had poor mental MSQOL-54 (P < 0.05).

Quality of life has been extensively studied in patients with multiple sclerosis [9]. Although research in this field is relatively new,the first papers appears in the literature in the early 1990s, it has been a subject of intense scientific research ever since [10]. Due to the progressive and disabling nature of the disease, Quality of life can be substantially reduced. In comparison to other studies which showed no significant relationship between QoL and the duration of disease [11]. Our research showed that, patients whom diagnosed with MS and have long disease duration had poor mental and physical health composite than patients with a shorter duration. In contrast others have found that, time since MS diagnosis have a significant effect on the mental aspect of quality of life [12] and it was differed across disease course but varied by age and duration of disease [13]. Furthermore, better quality of life score was reported in people with longer disease duration.

### Conclusion

The present study concludes that, multiple sclerosis disease among Sudanese patients was considerably affected in the Quality of life. Moreover; patients with advanced age, lower than university education and had longer disease duration was significantly associated with poor Physical Health Composite and Mental Health Composite.

## Limitation

The study was a cross-sectional study, so a large sample size was needed to consolidate our findings.

The questionnaire needs a translation to Arabic language. Examination of the responsiveness requires longitudinal data. The questionnaire is lengthy and need about 40 min to 1 h for each patient.

## Additional files

**Additional file 1.** MSQol-54 questionnaire.

**Additional file 2.** The comparison between MSQoL-54 subsets (PHC and MHC) in MS patients in Khartoum state regarding to the MS duration (N = 32).

## Data Availability

The datasets used and/or analyzed during the current study are available from the corresponding author on reasonable request.

## Abbreviations

HRQOL: health related quality of life
MHC: Mental Health Composite
MS: multiple sclerosis
MSQOL-54: multiple sclerosis quality of life-54 questionnaire
PHC: Physical Health Composite
QOL: quality of life
WHO: World Health Organization
