# Supplementary material for: Quality of life among Sudanese patients with multiple sclerosis in Khartoum state using (MSQoL-54) questionnaire
Source: BMC Res Notes. 2019 Aug 22;12:533. doi: 10.1186/s13104-019-4565-9 (PMC6704515; doi:10.1186/s13104-019-4565-9)
Supplement: Supplementary file 1 — Additional file 1. MSQol-54 questionnaire. [file 13104_2019_4565_MOESM1_ESM.pdf]

Multiple Sclerosis Quality of Life

(MSQOL)-54 Instrument

For Further Information, Contact:

Barbara G. Vickrey, MD, MPH
UCLA Department of Neurology
C-128 RNRC; Box 951769
Los Angeles, CA 90095-1769
Voice: 310.206.7671
Fax: 310.794.7716

Copyright© 1995, University of California, Los Angeles

INSTRUCTIONS:

This survey asks about your health and daily activities. Answer every question by circling the appropriate number (1, 2, 3, ...).

If you are unsure about how to answer a question, please give the best answer you can and write a comment or explanation in the margin.

Please feel free to ask someone to assist you if you need help reading or marking the form.

1. In general, would you say your health is:

(circle one number)

Excellent……………………………………1

Very good………………………………….2

Good………………………………………..3

Fair…………………………………..……...4

Poor…………………………………………5

2. **Compared to one year ago**, how would you rate your health in general **now**?

(circle one number)

Much better now than one year ago....................... 1

Somewhat better now than one year ago……….2

About the same 3

Somewhat worse now than one year ago 4

Much worse now than one year ago 5

Copyright© 1995, University of California, Los Angeles

3-12. The following questions are about activities you might do during a typical day. Does **your health** limit you in these activities? If so, how much?
(Circle 1, 2, or 3 on each line)

	Yes, Limited a Lot	Yes, Limited a Little	No, Not Limited at All
3. Vigorous activities, such as running, lifting heavy objects, participating in strenuous sports	1	2	3
4. Moderate activities, such as moving a table, pushing a vacuum cleaner, bowling, or playing golf	1	2	3
5. Lifting or carrying groceries	1	2	3
6. Climbing several flights of stairs	1	2	3
7. Climbing one flight of stairs	1	2	3
8. Bending, kneeling, or stooping	1	2	3
9. Walking more than a mile	1	2	3
10. Walking several blocks	1	2	3
11. Walking one block	1	2	3
12. Bathing and dressing yourself	1	2	3

Copyright© 1995, University of California, Los Angeles

13-16. During the **past 4 weeks**, have you had any of the following problems with your work or other regular daily activities **as a result of your physical health**?

(Circle one number on each line)

	YES	NO
13. Cut down on the amount of time you could spend on work or other activities	1	2
14. Accomplished less than you would like	1	2
15. Were limited in the kind of work or other activities	1	2
16. Had difficulty performing the work or other activities (for example, it took extra effort)	1	2

17-19. During the **past 4 weeks**, have you had any of the following problems with your work or other regular daily activities **as a result of any emotional problems** (such as feeling depressed or anxious).

(Circle one number on each line)

	YES	NO
17. Cut down on the amount of time you could spend on work or other activities	1	2
18. Accomplished less than you would like	1	2
19. Didn't do work or other activities as carefully as usual	1	2

Copyright© 1995, University of California, Los Angeles

20. During the **past 4 weeks**, to what extent has your physical health or emotional problems interfered with your normal social activities with family, friends, neighbors, or groups?

(circle one number)

Not at all..1

Slightly 2

Moderately.....................................3

Quite a bit 4

Extremely.......................................5

Pain

21. How much **bodily** pain have you had during the **past 4 weeks**?

(circle one number)

None 1

Very mild................................ 2

Mild 3

Moderate................................ 4

Severe 5

Very severe............................ 6

22. During the **past 4 weeks**, how much did **pain** interfere with your normal work (including both work outside the home and housework)?

(circle one number)

Not at all................................ 1

A little bit 2

Moderately............................ 3

Quite a bit 4

Extremely.............................. 5

Copyright© 1995, University of California, Los Angeles

23-32. These questions are about how you feel and how things have been with you **during the past 4 weeks**. For each question, please give the one answer that comes closest to the way you have been feeling.

How much of the time during the **past 4 weeks...** (Circle one number on each line)

	All of the Time	Most Of the Time	A Good Bit of the Time	Some of the Time	A Little of the Time	None of the Time
23. Did you feel full of pep?	1	2	3	4	5	6
24. Have you been a very nervous person?	1	2	3	4	5	6
25. Have you felt so down in the dumps that nothing could cheer you up?	1	2	3	4	5	6
26. Have you felt calm and peaceful?	1	2	3	4	5	6
27. Did you have a lot of energy?	1	2	3	4	5	6
28. Have you felt downhearted and blue?	1	2	3	4	5	6
29. Did you feel worn out?	1	2	3	4	5	6
30. Have you been a happy person?	1	2	3	4	5	6
31. Did you feel tired?	1	2	3	4	5	6
32. Did you feel rested on waking in the morning?	1	2	3	4	5	6

Copyright© 1995, University of California, Los Angeles

33. During the **past 4 weeks**, how much of the time has your **physical health or emotional problems** interfered with your social activities (like visiting with friends, relatives, etc.)?

(circle one number)

All of the time.........................1

Most of the time.....................2

Some of the time 3

A little of the time....................4

None of the time 5

Health in General

34-37. How TRUE or FALSE is each of the following statements for you.

(Circle one number on each line)

	Definitely True	Mostly True	Not Sure	Mostly False	Definitely False
34. I seem to get sick a little easier than other people	1	2	3	4	5
35. I am as healthy as anybody I know	1	2	3	4	5
36. I expect my health to get worse	1	2	3	4	5
37. My health is excellent	1	2	3	4	5

Copyright© 1995, University of California, Los Angeles

Health Distress

How much of the time during the **past 4 weeks...**

(Circle one number on each line)

	All of the Time	Most of the Time	A Good Bit of the Time	Some of the Time	A Little of the Time	None of the Time
38. Were you discouraged by your health problems?	1	2	3	4	5	6
39. Were you frustrated about your health?	1	2	3	4	5	6
40. Was your health a worry in your life?	1	2	3	4	5	6
41. Did you feel weighed down by your health problems?	1	2	3	4	5	6

Copyright© 1995, University of California, Los Angeles

Cognitive Function

How much of the time during the **past 4 weeks...**

(Circle one number on each line)

	All of the Time	Most of the Time	A Good Bit of the Time	Some of the Time	A Little of the Time	None of the Time
42. Have you had difficulty concentrating and thinking?	1	2	3	4	5	6
43. Did you have trouble keeping your attention on an activity for long?	1	2	3	4	5	6
44. Have you had trouble with your memory?	1	2	3	4	5	6
45. Have others, such as family members or friends, noticed that you have trouble with your memory or problems with your concentration?	1	2	3	4	5	6

Copyright© 1995, University of California, Los Angeles

Sexual Function

46-50. The next set of questions are about your sexual function and your satisfaction with your sexual function. Please answer as accurately as possible about your function **during the last 4 weeks only.**

How much of a problem was each of the following for you **during the past 4 weeks?**

(Circle one number on each line)

**MEN**	Not a problem	A Little of a Problem	Somewhat of a Problem	Very Much a Problem
46. Lack of sexual interest	1	2	3	4
47. Difficulty getting or keeping an erection	1	2	3	4
48. Difficulty having orgasm	1	2	3	4
49. Ability to satisfy sexual partner	1	2	3	4

(Circle one number on each line)

**WOMEN**	Not a problem	A Little of a Problem	Somewhat of a Problem	Very Much a Problem
46. Lack of sexual interest	1	2	3	4
47. Inadequate lubrication	1	2	3	4
48. Difficulty having orgasm	1	2	3	4
49. Ability to satisfy sexual partner	1	2	3	4

Copyright© 1995, University of California, Los Angeles

50. Overall, how satisfied were you with your sexual function **during the past 4 weeks**?

(circle one number)

Very satisfied.................................. 1

Somewhat satisfied........................ 2

Neither satisfied nor
dissatisfied 3

Somewhat dissatisfied 4

Very dissatisfied............................. 5

51. During the **past 4 weeks**, to what extent have problems with your bowel or bladder function interfered with your normal social activities with family, friends, neighbors, or groups?

(circle one number)

Not at all 1

Slightly.. 2

Moderately 3

Quite a bit....................................... 4

Extremely 5

52. During the **past 4 weeks**, how much did *pain* interfere with your enjoyment of life?

(circle one number)

Not at all 1

Slightly.. 2

Moderately 3

Quite a bit....................................... 4

Extremely 5

Copyright© 1995, University of California, Los Angeles

53. Overall, how would you rate your own quality-of-life?

Circle one number on the scale below:

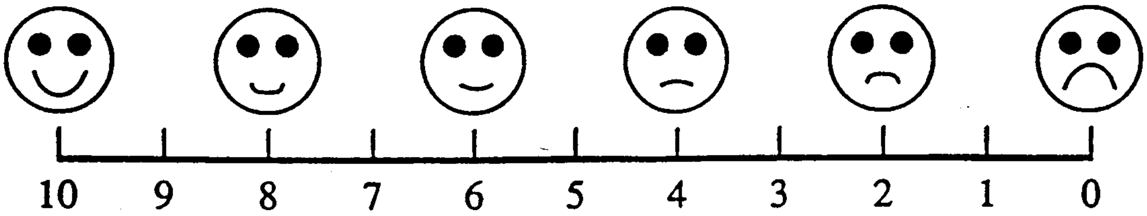

Best Possible Quality-of-Life

Worst Possible Quality-of-Life
As bad as or worse than being dead

54. Which best describes how you feel about your life as a whole?

(circle one number)

Terrible 1

Unhappy.. 2

Mostly dissatisfied 3

Mixed - about equally
satisfied and dissatisfied 4

Mostly satisfied............................... 5

Pleased 6

Delighted 7

Copyright© 1995, University of California, Los Angeles

Scoring Forms for Multiple Sclerosis Quality of Life (MSQOL) -54

Table 1
MSQOL-54 Scoring Form

Table 2
MSQOL-54 Physical Health Composite Score

Table 3
MSQOL-54 Mental Health Composite Score

Table 1

MSQOL-54 Scoring Form

**Scale/Item Number**	**Response 1**	**2**	**3**	**4**	**5**	**6**		**Subtotal**	**Final Score 0-100 point scale**
**Physical Health**									
3.	0	50	100					______	
4.	0	50	100					______	
5.	0	50	100					______	
6.	0	50	100					______	
7.	0	50	100					______	
8.	0	50	100					______	
9.	0	50	100					______	
10.	0	50	100					______	
11.	0	50	100					______	
12.	0	50	100					______	
							Total:	______ ÷ 10 =	______
**Role limitations due to physical problems**									
13.	0	100						______	
14.	0	100						______	
15.	0	100						______	
16.	0	100						______	
							Total:	______ ÷ 4 =	______
**Role limitations due to emotional problems**									
17.	0	100						______	
18.	0	100						______	
19.	0	100						______	
							Total:	______ ÷ 3 =	______
**Pain**									
21.	100	80	60	40	20	0		______	
22.	100	75	50	25	0			______	
52.	100	75	50	25	0			______	
							Total:	______ ÷ 3 =	______
**Emotional well-being**									
24.	0	20	40	60	80	100		______	
25.	0	20	40	60	80	100		______	
26.	100	80	60	40	20	0		______	
28.	0	20	40	60	80	100		______	
30.	100	80	60	40	20	0		______	
							Total:	______ ÷ 5 =	______
**Energy**									
23.	100	80	60	40	20	0		______	
27.	100	80	60	40	20	0		______	
29.	0	20	40	60	80	100		______	
31.	0	20	40	60	80	100		______	
32.	100	80	60	40	20	0		______	
							Total:	______ ÷ 5 =	______

Table 1 (cont.)

**Scale/Item Number**	**Response 1**	**2**	**3**	**4**	**5**	**6**	**Subtotal**	**Final Score 0-100 point**

Health Perceptions

1.	100	75	50	25	0			_____	
34.	0	25	50	75	100			_____	
35.	100	75	50	25	0			_____	
36.	0	25	50	75	100			_____	
37.	100	75	50	25	0			_____	
							Total:	_____ ÷ 5 =	_____

Social function

20.	100	75	50	25	0			_____	
33.	0	25	50	75	100			_____	
51.	100	75	50	25	0			_____	
							Total:	_____ ÷ 3 =	_____

Cognitive function

42.	0	20	40	60	80	100		_____	
43.	0	20	40	60	80	100		_____	
44.	0	20	40	60	80	100		_____	
45.	0	20	40	60	80	100		_____	
							Total:	_____ ÷ 4 =	_____

Health distress

38.	0	20	40	60	80	100		_____	
39.	0	20	40	60	80	100		_____	
40.	0	20	40	60	80	100		_____	
41.	0	20	40	60	80	100		_____	
							Total:	_____ ÷ 4 =	_____

Sexual function*

46.	100	66.7	33.3	0			_____	
47.	100	66.7	33.3	0			_____	
48.	100	66.7	33.3	0			_____	
49.	100	66.7	33.3	0			_____	
						Total:	_____ ÷ 4 =	_____

Change in health

2.	100	75	50	25	0	_____

Satisfaction with sexual function

50.	100	75	50	25	0	_____

	Response									
**Overall quality of life**	**1**	**2**	**3**	**4**	**5**	**6**	**7**			
53.	(multiply response by 10)								_____	
54.	0	16.7	33.3	50	66.7	83.3	100		_____	
								Total:	_____ ÷ 2 =	_____

Note: The total number of items in each scale is listed as the divisor for each subtotal. However, due to missing data, the divisor might actually be less than that if not every item within a given scale has been answered. For example, if item 38 in the Health Distress scale was left blank and the other 3 items in the scale were answered, then the "Total" score for Health Distress would be divided by '3' (instead of '4') to obtain the "Final Score."

* Males and females can be combined in the analysis even though question 47 is different for the two groups. The scale scores can also be reported separately for males and females.

Table 2
Formula for calculating MSQOL-54 Physical Health Composite Score

MSQOL-54 Scale	Final Scale Score	x	Weight	=	Subtotal
Physical function	_____	x	.17	=	_____(a)
Health perceptions	_____	x	.17	=	_____(b)
Energy/fatigue	_____	x	.12	=	_____(c)
Role limitations - physical	_____	x	.12	=	_____(d)
Pain	_____	x	.11	=	_____(e)
Sexual function	_____	x	.08	=	_____(f)
Social function	_____	x	.12	=	_____(g)
Health distress	_____	x	.11	=	_____(h)

PHYSICAL HEALTH COMPOSITE: Sum subtotals (a) through (h) = _____

Table 3
Formula for calculating MSQOL-54 Mental Health Composite Score

MSQOL-54 Scale	Final Scale Score	x	Weight	=	Subtotal
Health distress	_____	x	.14	=	_____(a)
Overall quality of life	_____	x	.18	=	_____(b)
Emotional well-being	_____	x	.29	=	_____(c)
Role limitations - emotional	_____	x	.24	=	_____(d)
Cognitive function	_____	x	.15	=	_____(e)

MENTAL HEALTH COMPOSITE: Sum subtotals (a) through (e) = _____
