# Supplementary material for: Quality of life among Sudanese patients with multiple sclerosis in Khartoum state using (MSQoL-54) questionnaire
Source: BMC Res Notes. 2019 Aug 22;12:533. doi: 10.1186/s13104-019-4565-9 (PMC6704515; doi:10.1186/s13104-019-4565-9)
Supplement: Supplementary file 2 — Additional file 2. The comparison between MSQoL-54 subsets (PHC and MHC) in MS patients in Khartoum state regarding to the MS duration (N = 32). [file 13104_2019_4565_MOESM2_ESM.docx]

**Table S1 shows** the comparison between MSQoL-54 subsets (PHC and MHC) in MS patients in Khartoum state regarding to the MS duration (N= 32)

| MS duration (years) | Physical Health Composite (PHC) | Mental Health Composite (MHC) |
| --- | --- | --- |
|  | Mean (SD) | Mean (SD) |
| <5 | 70.2 (14.1) | 75.3 (13.6) |
| 5-10 | 60.8 (19.8) | 66.7 (14.3) |
| >10 | 51.1 (12.2) | 58.8 (12.7) |
| P. value | 0.000* | 0.000* |
| ANOVA test was used  *P. value is significant (< 0.05) | | |
